# Parkinson patients without tremor show changed patterns of mechanical muscle oscillations during a specific bilateral motor task compared to controls

**DOI:** 10.1038/s41598-020-57766-5

**Published:** 2020-01-24

**Authors:** Laura V. Schaefer, Frank N. Bittmann

**Affiliations:** Regulative Physiology and Prevention, Department Sports and Health Sciences, University of Potsdam, Potsdam, Germany

**Keywords:** Parkinson's disease, Parkinson's disease, Parkinson's disease, Motor control, Neurophysiology

## Abstract

The pathophysiology of Parkinson’s disease (PD) is still not understood. There are investigations which show a changed oscillatory behaviour of brain circuits or changes in variability of, e.g., gait parameters in PD. The aim of this study was to investigate whether or not the motor output differs between PD patients and healthy controls. Thereby, patients without tremor are investigated in the medication off state performing a special bilateral isometric motor task. The force and accelerations (ACC) were recorded as well as the Mechanomyography (MMG) of the biceps brachii, the brachioradialis and of the pectoralis major muscles using piezoelectric-sensors during the bilateral motor task at 60% of the maximal isometric contraction. The frequency, a specific power ratio, the amplitude variation and the slope of amplitudes were analysed. The results indicate that the oscillatory behaviour of motor output in PD patients without tremor deviates from controls: thereby, the 95%-confidence-intervals of power ratio and of amplitude variation of all signals are disjoint between PD and controls and show significant differences in group comparisons (power ratio: p = 0.000–0.004, r = 0.441–0.579; amplitude variation: p = 0.000–0.001, r = 0.37–0.67). The mean frequency shows a significant difference for ACC (p = 0.009, r = 0.43), but not for MMG. It remains open, whether this muscular output reflects changes of brain circuits and whether the results are reproducible and specific for PD.

## Introduction

The pathogenesis and pathomechanism of Parkinson’s disease (PD) are not clarified yet^1–4^. A damage or death of neuronal cells through aggregation of misfolded alpha-synuclein in Lewy bodies is reported to be necessary^5,6^. The occurrence of those Lewy bodies in PD are reported for brain and other nerval structures as the spinal cord, peripheral nervous system, cervical sympathetic trunk and vagal nerve^6^, but also for the submandibular gland or the upper gastrointestinal tract^4^. The misfolded alpha-synuclein spread in diverse cells and lead to several deficits of bodily functions as e.g. mitochondrial function^5,6^. However, also other proteins than alpha-synuclein and other pathological changes are discussed to be relevant in PD^6,7^. This emphasises the complexity of PD and explains the different symptom appearances in patients, which complicates the diagnosis.

PD is characterized by the presence of multiple motor and nonmotor symptoms^1,6,7^. The motor symptoms are not limited to tremor, but also appear as postural instability or gait impairment^6,8,9^. In addition, the tremor appears not only as resting tremor, but also as holding or postural tremor^10^. The motor symptoms are primarily explained by the loss of dopaminergic cells in the substantia nigra^2,5–7^. If the motor stage is reached, already around 50% of the dopaminergic neurons of the total substantia nigra are lost^11,12^ and around 80% of the striatal dopamine is reduced^12–14^. If the amount of loss is lower, the patients are in the so called premotor state. Especially in the early stages, the classic cardinal symptoms bradykinesia, rigidity and resting tremor are often so inconspicuous that a clinical diagnosis becomes difficult. Therefore, a challenge arises from the task of identifying persons at risk already in this stage. It can be assumed that even in the preclinical phase slight motor abnormalities develop, which are not obvious to the observer and the person concerned^1^. Besides, a substantial proportion of PD patients are misdiagnosed^15–17^.

Until now, the diagnosis is primarily based on clinical findings using the Unified Parkinson’s Disease Rating Scale (UPDRS)^5,10^, which can be supplemented by various additional examinations (e.g. single photon emission computed tomography, midbrain sonography, odour test, polysomnography). The diagnosis is further complicated since atypical parkinsonian syndromes (aPS), including progressive supranuclear palsy, multiple system atrophy and also corticobasal degeneration, often mimic PD for the first few years^17,18^.

An early diagnosis is decisively important, since treatment initiation should start as early as possible^1^ to decelerate the further loss of dopaminergic cells and to achieve a reduction in symptoms and the delay of levodopa initiation^19^. Although there are various approaches to investigate promising biomarkers, Kalia and Lang^6^ state that no sufficient objective diagnostic tool exists. They give an overview of currently investigated potential biomarkers for diagnosis of PD^6^. A biomarker concerning pathological changes in neuromuscular oscillations is not considered therein.

However, in investigating the pathophysiology of motor symptoms of PD, the neuronal oscillations of brain activity are in the focus of interest, especially with respect to the basal-ganglia circuitry^14,20,21^. Thereby, it is assumed that not the excitatory or inhibitory degree but rather the patterning of the basal-ganglia-cortical loop activities lead to typical symptoms^21^. In particular, a synchronisation of basal-ganglia-cortical loop oscillations in the beta-band (13–30 Hz) is found^21^. Furthermore, changes in the patterns of high frequency oscillations of subthalamic nucleus are reported^22–24^. Assuming that these altered patterns of the motor controlling brain activity might occur already in early stage PD, it would be conceivable to expect pattern changes in the muscular activity.

EMG is used in standard clinical practice to monitor the electrical motor output and to get more insights into the changes in PD. However, it could not be developed into a supportive diagnostic tool in PD up to now^6,25,26^. The found changes in EMG are mostly interpreted to refer to the resting tremor^26^. For example, Rissanen *et al*.^27^ tried to cluster PD patients and healthy controls using the signals of acceleration and 12 EMGs. The clustering was sensitive for tremor. Thus, the patients with tremor could be discriminated from the controls. However, the ten PD patients “with only little or no tremor at all were clustered into the cluster of mostly healthy persons”^27^. This underlines the difficulty in diagnosing patients without tremor and, probably, also the disadavantages of evaluating EMG signals in PD.

Another method for detecting the motor output is the Mechanomyography (MMG). MMG is considered as the mechanical counterpart of EMG and capture the mechanical muscular micro-oscillations^28,29^. The synchronized oscillation of several thousand fibres of a muscle indicates the tremendous integration power of the sensorimotor neurostructures which are responsible for the intramuscular coordination. It is assumed that the mechanical muscle oscillations could be vulnerable for changes in motor control.

Marusiak *et al*.^29–32^ already have performed measurements using MMG in Parkinson’s disease. They recorded MMG and EMG from the elbow flexor and extensor muscles during submaximal isometric muscle action in Parkinson patients with tremor^30^. They found differences between healthy controls and Parkinson patients and suggested that MMG – also with regard to EMG – is a valuable tool for investigating neuromuscular diseases as Parkinson’s^30^. Since the patients already had a visible tremor, the result of a lower median frequency in MMG is not surprising. If the clinical tremor already is visible, EMG, MMG and acceleration sensors are able to record those macro-oscillations.

Therefore, in order to monitor changes in premotor stages, Parkinson patients without tremor should be considered.

With the overarching aim to contribute to the diagnosis of PD especially in the early stages, the question arises whether or not the oscillatory patterns of mechanical muscular oscillations already show changes in Parkinson’s patients without a clinical tremor.

## Methods

The aim of this exploratory study was to investigate how the mechanical myofascial oscillations behave in two different tasks of isometric muscle action in patients with PD without tremor compared to healthy controls. The feature of the study is that PD patients without tremor are investigated in a unilateral and a specific bilateral task, whereby the subject is interacting with itself. Because of the complexity of the study only the setting and results of the bilateral task is presented in this article. The unilateral task will be reported later. The study was funded by the German Society for Parkinson’s and movement disorders.

### Participants

#### Parkinson patients

A total of n = 28 patients with PD *without a tremor* (PD) volunteered to participate in the study. As part of the clinical routine, measurements were performed during the medication off phase. The patients were recruited from the Neurological Clinic for Movement Disorders and Parkinson’s Disease in Beelitz-Heilstätten (Germany; Chief physician: Prof. Dr. G. Ebersbach) and were diagnosed with PD without the appearance of a tremor. Exclusion criteria were the appearance of a clinical tremor, neurological symptoms beyond PD, a manifest polyneuropathy, a coronary heart disease of NYHA III or higher, brain pacemaker, brain aneurysms, glaucoma and haemorrhagic apoplexy. A relative exclusion criteria was orthopaedic symptoms of the upper extremities, the shoulder girdle and cervical spine within the last six months before the measurement.

In total, n = 10 patients had to be excluded because of complaints during the measurements (n = 3), because of reduced signal quality (n = 4) or other factors (n = 3). The anthropometric data and the bilaterally measured maximal voluntary isometric contraction (bMVIC) are displayed in Table 1.

**Table 1:** Anthropometric data and averaged values of the bilateral maximal voluntary isometric contraction (bMVIC) of all included subjects. Displayed are the arithmetic mean and standard deviation.

Gender	PD patients		Controls	
	m	f	m	f
n	14	4	11	12
Age [years]	58.80 ± 18.83	68.75 ± 9.81	70.00 ± 6.26	66.09 ± 7.61
Body mass [kg]	84.66 ± 17.05	71.00 ± 23.76	77.30 ± 7.72	71.36 ± 7.93
Body height [cm]	177.46 ± 7.05	162.50 ± 5.20	176.00 ± 6.01	164.82 ± 6.57
bMVIC [Nm]	54.96 ± 23.76	25.85 ± 7.83	72.20 ± 29.17	30.54 ± 11.81

### Controls

A total of n = 29 healthy subjects (Con) volunteered to participate in the study. In total six participants had to be excluded: due to the neurological examination (n = 4), detaching of sensor fixation caused by sweating (heat) (n = 1) or due to reduced signal quality (n = 1), respectively. The remaining n = 23 healthy subjects (m = 11, f = 12) were recruited from the ‘Club Aktiv’ of the Brandenburgischer Verein für Gesundheitsförderung e.V. (BVfG; Brandenburg Association of Health Promotion) or from relatives of the patients. The anthropometric data and the bMVIC of the upper extremity are displayed in Table 1. Exclusion criteria were complaints of the upper extremities, the shoulder girdle and cervical spine within the last six months before the measurement and any hints for a neurological disease. The neurological examinations were performed by a neurologist (specialist for movement disorders and PD) from the Neurological Clinic for Movement Disorders and Parkinson’s Disease (Beelitz-Heilstätten, Germany).

### Setting

**Figure 1:**
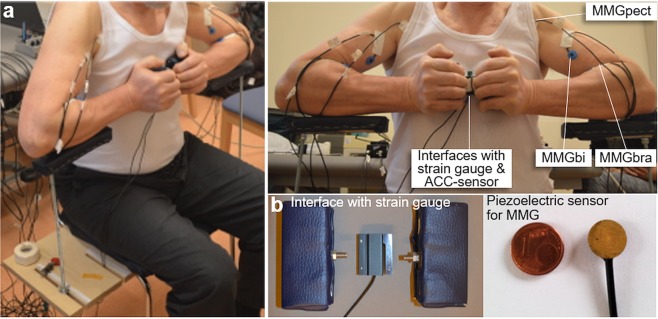
Setting of the bilateral motor task. (**a**) Measuring position for recording the acceleration, force and mechanical muscular oscillations (MMGs) of the biceps brachii, brachioradialis and pectoralis major muscles during the bilateral isometric muscle action. The subject had to push its hands from each side against the interface and intrapersonal reaction force of both arms. (**b**) Illustrates the strain gauge with interfaces for the bilateral task and the piezoelectric sensor for recording the MMGs.

Figure 1a illustrates the measuring position of the bilateral motor task. This task was chosen in particular because, firstly, it is more complex than contracting only one side, but still is easy to perform for the patients. Secondly, there is a lot of experience concerning the reliability using MMG to measure muscle activity of the upper extremity during isometric muscle action^28,33,34^.

The subject sat on a custom-made chair with 90° hip- and knee angle and held the interface including a strain gauge (model: ML MZ 2000 N 78, 2000N, modified by biovision; Fig. 1b) between its hands in front of its thorax. By pressing the interfaces against each other, the reaction force between both hands could be recorded. An acceleration sensor (ACC) with a sensitivity of 312 mV/g (range ± 2 g, linearity: ± 0.2%; comp.: biovision) was fixed at the strain gauge to detect the accelerations along the longitudinal acting force vector. The arms were not fixed, thus, could oscillate freely.

The mechanical oscillations of the biceps brachii (MMGbi), the brachioradialis (MMGbra) and the pectoralis major muscles (MMGpect) were recorded using piezoelectric sensors (model: Shadow SH 4001). The sensors were fixed above the muscle bellies, respectively, with a special tape originally used for ECGs. To identify the proper position for the sensors, the patients should take up the measurement position and briefly activate the muscles. The sensor was fixed at the area with maximal muscle tension.

The MMG-signals were amplified using the Nobels preamp booster pre-1 (adjustments: Bass: 9, Treble: 5, Level: 9). All signals were conducted to an A/D-converter (National Instruments, 14-bit, USB-6009; modified by Biovision) and subsequently were recorded by the software NI DIAdem 2012 (National Instruments) on a measurement notebook (Toshiba Satellite Pro L500-1T2; Windows 7). Sampling rate was set at 1000 Hz.

### Measuring procedure

The subjects received an information letter, in which the procedure and setting was explained in detail, at least one day before experiment. The patients were examined by neurologists of the Neurological Clinic for Movement Disorders and Parkinson’s Disease (Beelitz-Heilstätten, Germany) using the UPDRS prior to the measuring on the experiment day. The controls were examined neurologically of one neurologist during a separate appointment. After examination, the subject was introduced to the system and procedure on site. Afterwards, the sensors were fixed and the subject took place on the chair. Generally, task I (unilateral) and II (bilateral) were performed subsequently. Firstly, task I was performed randomized for the left and right side (9 trials: 1 x resting position, 1 x starting position, 2 x MVIC, 5 × 70% of MVIC). These trials will not be presented here. Since they may have influenced the subsequent bilateral task, they have to be mentioned here. The 9 trials of the bilateral motor task followed: Firstly, a resting position was chosen to indicate a still probably existing resting tremor. Thereby, the subject had its hands on lap. Afterwards, the subject performed two maximal contractions in the bilateral setting: Thereby, it should push its hands maximally against the interfaces to identify the maximal bilateral voluntary isometric contraction (bMVIC). This represents the maximal reaction force between left and right upper extremity. The further five trials were performed in the same setting with an intensity of 60% of the bMVIC, which was calculated by using the highest value of the two bMVIC-trials. The intensity of 60% of bMVIC should be maintained for 10 s. The last trial was performed in a resting position again. The resting period between each trial was set at 90 s.

### Data processing and statistical analysis

**Figure 2:**
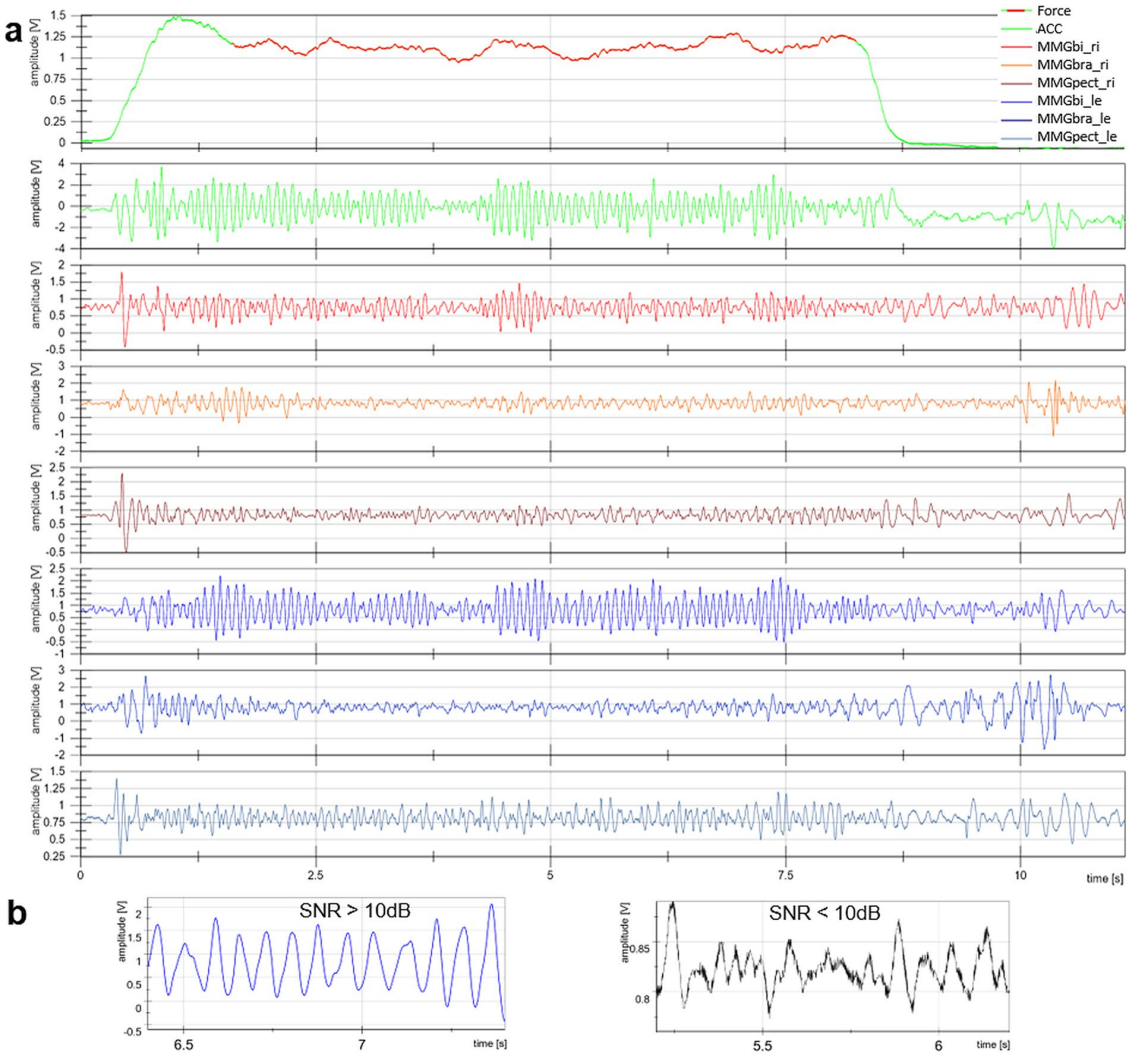
Exemplary raw signals. (**a**) The upper six panels display the raw signals of force, ACC, MMGbi, MMGbra, MMGpect of right (upper three panels) and left side (lower three panels) of a PD patient during one trial of the bilateral task. The red area of the force signal indicates the cut out isometric plateau. (**b**) The panels at the bottom show exemplary raw signals with very good (blue, left; SNR > 10 dB) and not accepted (black, right; SNR < 10 dB) SNR.

NI DIAdem 14 was used for the data processing and in parts for the analysis. For further considerations Excel (Microsoft Office, 2013) was utilized and SPSS Statistics 25 (IBM) was applied for statistical comparisons. A comparison with the UPDRS was not done in this evaluation. Concerning the analysis of oscillating signals, a signal-noise-ratio (SNR) of 10 dB was provided. Raw signals with a lower SNR were excluded. Exemplary raw signals are displayed in Fig. 2.

For the group comparisons between PD and Con the following parameters were considered (detailed description see below): (1) bMVIC (force signal); and parameters for the oscillating signals MMGs and ACC: (2) arithmetic mean (M) and coefficient of variation (CV) of a specific power ratio (Q_REL_) of the Power Spectral Density (PSD); (3) the slope of the amplitude maxima, (4) the variation of the amplitudes in-between one trial and (5) the mean frequency. For the parameters (2)-(5), the isometric plateau of the five trials at 60% of the bMVIC was cut from the raw data (from force signal; deviations of ± 10% were accepted) and was used for further considerations.

#### Bilateral maximal voluntary isometric contraction (bMVIC)

The maximum cursor was used in NI DIAdem to determine the maximal value of the filtered force signal (filter type: Butterworth; filtering degree 10, cut-off frequency 3). The higher value of both bMVIC trials was transmitted in Excel and SPSS for group comparisons.

#### Specific power ratio Q_REL_

This specific power ratio was evolved exploratively in a pilot study and was marginally adjusted for this investigation. The idea behind this parameter is to get the percentage of the power in the low frequency range of 3 to 7 Hz on the power in the wider frequency range of 3 to 12 Hz. In the pilot study the PSD of Parkinson patients indicated that two main peaks exists: one in the physiological frequency range of around 10–15 Hz and another in a lower frequency range of around 5 Hz. Considering only the mean frequency would level the two peaks.

**Figure 3:**
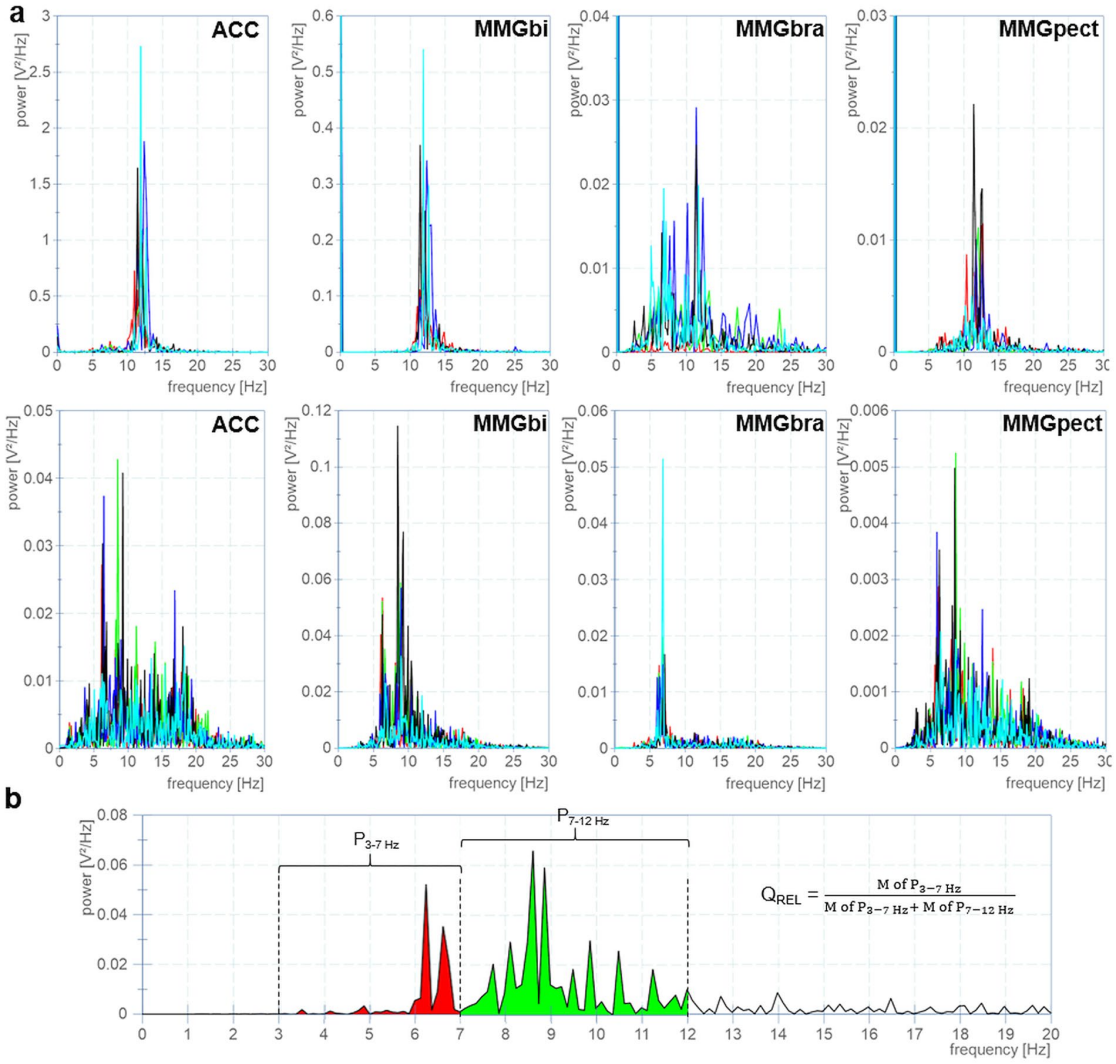
Exemplary power density spectra of oscillating signals. (**a**) The four panels above display the PSD of each five trials of the ACC, MMGbi, MMGbra and MMGpect of one PD patient (left side) during the bilateral task. The four panels in the middle show the same for a healthy control. (**b**) Exemplary power density spectra of one MMGbi signal of a healthy control including the analysis of the power ratio Q_REL_. The proportion of the arithmetic mean (M) of power (P) in the Interval 1 (P_3-7 Hz_; red) to the sum of the arithmetic means of power in the frequency band of 3 to 7 Hz (red) and 7 to 12 Hz (P_7–12 Hz_; green) is the parameter of interest.

The raw data were used to determine Q_REL_, which is calculated using the values of the PSD (Fig. 3):

$${Q}_{REL}=\frac{{\rm{M}}\,{\rm{of}}\,{\rm{power}}\,{\rm{in}}\,{\rm{the}}\,{\rm{frequency}}\,{\rm{range}}\,{\rm{of}}\,3\,{\rm{to}}\,7\,{\rm{Hz}}}{({\rm{M}}\,{\rm{of}}\,{\rm{power}}\,{\rm{in}}\,{\rm{frequency}}\,{\rm{range}}\,{\rm{of}}\,3\,{\rm{to}}\,7\,\text{Hz})+({\rm{M}}\,{\rm{of}}\,{\rm{power}}\,{\rm{in}}\,{\rm{frequency}}\,{\rm{range}}\,{\rm{of}}\,7\,{\rm{to}}\,12\,\text{Hz})}$$

**Figure 4:**
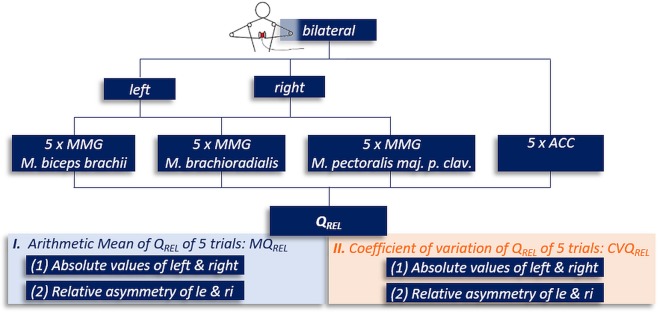
Overview of specific parameters concerning the power ratio Q_REL_. The considered specific parameters concerning the power ratio Q_REL_ of the PSDs of the MMGs- and ACC-signals are displayed. The MMG-signals were recorded from the left and right side. Only one ACC-signal exists in the bilateral task. Main parameters are the arithmetic mean and coefficient of variation of Q_REL_, considered separately for each side and with regard to side asymmetries (Diff).

For each trial and each MMG-/ACC-signal one value Q_REL_ results. For further investigations and analysis the following variables were calculated out of the five power ratios Q_REL_ (Fig. 4):

I. Arithmetic mean (M) of the five Q_REL_ for each signal (MMGs, ACC) and side:

1. **MQ**_**REL**_ = absolute value of MQ_REL_
2. Relative asymmetry of left (le) and right (ri) side of MQ_REL_: (not applicable for ACC)

$${\bf{Diff}} \mbox{-} {\bf{M}}{{\bf{Q}}}_{{\bf{R}}{\bf{E}}{\bf{L}}}=\left|\left(\frac{M{Q}_{REL}le}{M{Q}_{REL}le+M{Q}_{REL}ri}\cdot 100\right)-50\right|$$

II. Coefficient of variation (CV) of the five Q_REL_ for each signal (MMGs, ACC) and side:

1. CVQ_REL_ = absolute value of the CV
2. Diff-CVQ_REL_ = relative asymmetry of left and right side of CVQ_REL_ (not applicable for ACC) (analogues to I.2)

Analysing the sum of power or the integral of PSD would lead to similar results.

### Data processing for parameters (3)-(5)

For the following parameters, all signals were filtered (Butterworth, filtering degree 5, cut-off frequency 20) and the maxima of the oscillations were determined.

#### Slope of the amplitude maxima

To calculate the mean slope of all maxima of one signal, the slope function in Excel was used. For each signal one slope value resulted per trial. In order to create a linear parameter for further consideration, this was converted into degree. Per subject and side five slope values resulted.

#### Amplitude variation (VAmp)

For the amplitude variation within one trial, the absolute difference between the y-values of each two consecutive maxima of one trial was calculated in Excel. The resulting differences were averaged per trial and were relativized to the arithmetic mean of the amplitudes (M of y-values of maxima). One value VAmp resulted per trial for each signal.

#### Mean frequency

The frequency of one signal was calculated by, firstly, determining the reciprocal of the time distances (x-values) of two consecutive maximum points and, secondly, averaging these values of one signal. For each signal one value of frequency resulted per trial. This method is rather unusual for stochastically distributed variables as captured here. However, concerning^35^ it is possible to use the reciprocal of period duration in almost periodic oscillations in chaotic systems, as the neuromuscular system. The authors regard this technique as appropriate for the present investigation to get information about the mean frequency, considering that, thereby, the amplitudes are not taken into account. The latter is done by parameter (2).

For further statistical analysis of the parameters (3), (4) and (5), the arithmetic mean (M) and coefficient of variation (${\rm{CV}}=\frac{Standard\,deviation\,(SD)}{M}$) were calculated for the signals MMGbi, MMGbra, MMGpect (left and right) and ACC using the values of the five trials. Furthermore, the relative asymmetry (Diff) of the left and the right side was calculated for MMGbi, MMGbra and MMGpect to show interlimb asymmetries.

### Statistical considerations

For the group statistics the data of each parameter and each group (PD vs. Con) were examined concerning normal distribution by means of Shapiro-Wilk-test. For the group comparisons of anthropometric data and bMVIC a one-way ANOVA was performed. For the above mentioned parameters an unpaired t-test for parametric data or the Mann-Whitney-U-test for non-parametric data were utilized to compare the groups PD and Con. For ANOVA and t-test the Levène test of variance homogeneity was performed and required. If variance homogeneity was not fulfilled, a Welch correction was performed. The gender differences were analysed using the Chi-squared test.

The effect size was determined by either the Pearson’s correlation coefficient r for parametric data or by the Cohens d for non-parametric data. Furthermore, the 95%-confidence intervals (CI) were calculated for the parameters (2)-(5) using the formula $CI=M\pm 1.96\left(\frac{SD}{\sqrt{n}}\right)$, whereby M is the arithmetic mean, SD is the standard deviation and n stands for the sample size. The value 1.96 stands for the z-value using a significance level of α = 0.05.

In general, the significance level was set at α = 0.05.

### Ethical approval

The study was approved by the ethic committee of the University of Potsdam and by the State Chamber of Medicine in Brandenburg, Germany. It was conducted in accordance with the declaration of Helsinki. All participants were informed in detail and gave their informed written consent to participate. The participant seen in Fig. 1 gave informed consent to publish both images in an online open acess publication.

## Results

The results of Shapiro Wilk test for normal distribution are provided in the supplementary material. According to those, the tests for group comparison were chosen.

### Anthropometric data and bMVIC

The anthropometric data show no significant differences between PD and Con regarding age (F(3,37) = 1.130; p = 0.349) and BMI (F(3,34) = 0.726, p = 0.543) (Table 1). The proportion of male and female in PD and Con groups shows no statistical difference taking all four groups into account (PD_m_ = 14, PD_f_ = 4, Con_m_ = 11, Con_f_ = 12; χ² = 5.537, p = 0.136). Looking only at the females, the proportion between the groups is statistically different (χ² = 4.0, p = 0.046).

As displayed in Table 1, the arithmetic mean of the bMVIC of male PD is about 27.8% lower compared to the Con group (n.s.; Bonferroni test of ANOVA: p = 0.125). Female PD show an approximately 15.4% lower bMVIC compared to Con (n.s.; Bonferroni test of ANOVA: p = 1.0). The ANOVA shows significant differences of bMVIC concerning the four groups PD male vs. female and control male vs. female, respectively (F(3,37) = 10.345, p = 0.000). The post-hoc Bonferroni-test displays no significant difference between the groups of PD and Con within the females and males, respectively (female: p = 1.0; male: p = 0.125). The bMVIC of males and females show significant differences in ANOVA, which are not relevant here.

### Specific power ratio Q_REL_

The statistical results of Q_REL_ (parameter (2)) of all signals are displayed in Table 2.

**Table 2:** Statistical values of parameters of Q_REL_ for each signal.

	MMGbi		MMGbra		MMGpect		ACC	
	PD	Con	PD	Con	PD	Con	PD	Con
Sample size n	29	38	27	30	27	38	13	21
MQ_REL_[Hz/V²]	0.20 ± 0.11	0.36 ± 0.17	0.26 ± 0.16	0.42 ± 0.17	0.18 ± 0.13	0.39 ± 0.15	0.16 ± 0.12	0.35 ± 0.24
Sign p (effect r)	0.000* (0.516)		**0.001 (0.441)**		**0.000 (0.579)**		0.004* (0.485)	
CVQ_REL_ [%]	35 ± 25	29 ± 17	30 ± 15	23 ± 11	31 ± 16	29 ± 14	29 ± 11	25 ± 11
Sign p	0.179		0.082		0.821		0.297	
Diff-MQ_REL_	13.9 ± 11.3	10.6 ± 8.6	12.5 ± 9.2	9.7 ± 5.7	7.3 ± 5.7	15.9 ± 10.2	—	
Sign p (effect r)	0.356		0.330		0.014* (0.547)		—	
Diff-CVQ_REL_	15.9 ± 9.6	13.5 ± 7.3	12.8 ± 10.7	12.8 ± 8.6	14.8 ± 7.0	13.7 ± 7.2	—	
Sign p	0.457		0.991		0.657		—	

Displayed are the arithmetic mean and SD, significance p of t-test for independent variables and effect size r of MQ_REL_ and CVQ_REL_ and their side asymmetries (Diff-MQ_REL_; Diff-CVQ_REL_ comparing PD and Con regarding the signals MMGbi, MMGbra, MMGpect and ACC.

*p-value*s are adjusted using Welch-correction*.

#### MMG-signals

**Figure 5:**
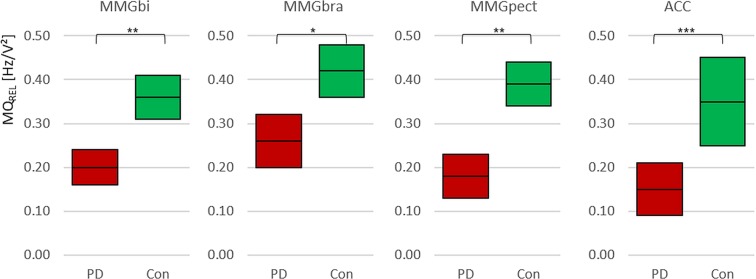
Specific Ratio MQ_REL_. Displayed are the 95%-confidence intervals of the arithmetic mean of the specific ratio Q_rel_ (MQ_REL_) of the MMG- and ACC-signals. As can be seen, the PD have a lower ratio compared to controls. Especially, the MMGbi and the MMGpect show similar endpoints of CI and high significant differences of ***p = 0.000. The MMGbra is still highly significant between PD and Con with **p = 0.001. The MQ_REL_ of ACC-signals varies more, especially in Con, but still is significant in comparing both groups (*p_adj_ = 0.004).

The 95%-confidence intervals of the variable MQ_REL_ for the MMG-signals of each muscle are displayed in Fig. 5. The MQ_REL_ of MMGbi and MMGpect show similar endpoints of 95%-CI and a highly significant difference in t-test for independent variables (MMGbi: t(65) = 4.550, p = 0.000, r = 0.49; MMGpect: t(63) = 5.635, p = 0.000, r = 0.58). The endpoints of the 95%-CI for the MMGbra are still disjoint and significant (t(55) = 3.641, p = 0.001, r = 0.44), but show higher endpoints compared to MMGbi and MMGpect (Fig. 5).

**Figure 6:**
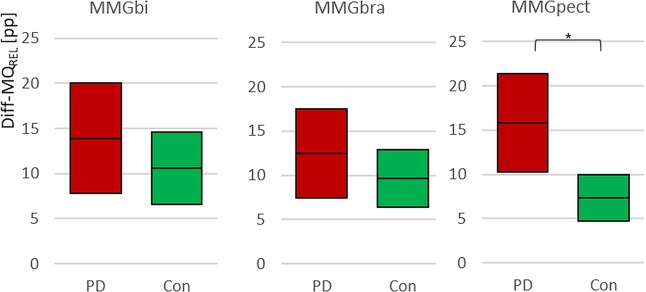
Side asymmetry of MQ_REL_. Displayed are the 95%-confidence intervals of the side asymmetry of the arithmetic mean values of the specific ratio Q_REL_ (Diff-MQ_REL_ [pp]) of the MMG-signals. As can be seen, the PD tend to have higher values of Diff-MQ_REL_, but only the MMGpect shows a significant difference between Con and PD. *p_adj_ = 0.014.

The relative side asymmetry between left and right of the MQ_REL_ (Diff-MQ_REL,_ Fig. 6) of the MMG-signals during bilateral task shows no significant difference between PD and Con for MMGbi and MMGbra, respectively, but for the MMGpect (MMGbi: t(29) = −0.939, p = 0.356; n_PD_ = 13; n_con_ = 18; MMGbra: t(21) = −0.998, p = 0.330; n_PD_ = 13; n_con_ = 12; MMGpect: t_adj_(17.45) = − 2.726, p_adj_ = 0.014, r = 0.55; n_PD_ = 13; n_con_ = 18). Thereby, the side difference is higher in PD patients.

The coefficient of variation of Q_REL_ shows no significant difference in the bilateral task, neither regarding CVQ_REL_ nor regarding the side asymmetry (Diff-CVQ_REL_) (Table 2).

#### ACC-signal

The 95%-CIs concerning the parameter MQ_REL_ of the ACC-signal are disjoint and significantly different between PD and Con (Table 2; Fig. 5; t_adj_(30.87) = 3.085, p_adj_ = 0.004, r = 0.49). The CVQ_REL_ of ACC-signal shows no significance between the groups (t(32) = −1.06, p = 0.297; Table 2).

### Further parameters of oscillatory signals

The M and CV of the parameters slope (3), amplitude variation (Vamp; (4)) and mean frequency (5) of the signals MMGbi, MMGbra, MMGpect and ACC are displayed in Table 3.

**Table 3:** M ± SD of the oscillatory parameters of the ACC- and MMG-signals of the less and more affected side of PD and the left and right side of Con, respectively, and the corresponding significance p and effect size r of the group comparisons between PD and Con.

ACC		PD (n = 16)		Con (n = 20)		Sign. p	Effect r
Slope [°]	M	0.32 ± 1.61		0.60 ± 1.50		0.671	—
	CV	1.82 ± 2.26		5.15 ± 2.23		0.741	—
VAmp	M	0.57 ± 0.13		0.83 ± 0.16		0.000	0.67
	CV	0.16 ± 0.04		0.11 ± 0.04		0.002	0.50
Freq [Hz]	M	12.99 ± 1.11		14.20 ± 1.28		0.009	0.43
	CV	0.038 ± 0.02		0.043 ± 0.02		0.912	—
**MMGbi (n**_**PD**_** = 37; n**_**Con **_**= 46)**		**PD**		**Con**		**Sign. p**	**Effect r**
		**Less affected**	**more affected**	**le**	**ri**		
Slope [°]	M	0.25 ± 0.34	0.29 ± 0.61	0.38 ± 0.53	0.34 ± 0.71	0.996	—
	CV	2.71 ± 3.35	1.69 ± 1.79	2.81 ± 6.69	3.29 ± 4.42	0.707	—
	DiffM	0.59 ± 0.69		0.59 ± 0.51		0.610	—
	DiffCV	19.83 ± 13.67		18.49 ± 13.14		0.699	—
VAmp	M	0.72 ± 0.17	0.73 ± 0.20	0.84 ± 0.10	0.88 ± 0.19	0.001	0.37
	CV	0.13 ± 0.08	0.16 ± 0.07	0.14 ± 0.06	0.15 ± 0.07	0.927	—
	DiffM	5.71 ± 2.93		5.18 ± 2.28		0.079	—
	DiffCV	13.38 ± 8.11		10.74 ± 8.65		0.284	—
Frequency [Hz]	M	13.94 ± 1.01	13.69 ± 1.16	14.46 ± 1.0	14.06 ± 1.5	0.105	—
	CV	0.05 ± 0.01	0.05 ± 0.02	0.05 ± 0.03	0.05 ± 0.04	0.847	—
	DiffM	1.88 ± 0.75		2.64 ± 1.78		0.394	—
	DiffCV	9.77 ± 7.99		11.41 ± 7.34		0.639	—
**MMGbra (n**_**PD**_** = 27; n**_**Con**_** = 30)**		**Less affected**	**more affected**	**le**	**ri**	**Sign. p**	**Effect r**
Slope [°]	M	0.18 ± 0.25	0.35 ± 0.58	0.004 ± 0.008	0.01 ± 0.18	0.749	—
	CV	1.42 ± 1.38	3.75 ± 8.73	3.65 ± 3.28	2.27 ± 3.21	0.152	—
	DiffM	0.323 ± 0.41		0.61 ± 1.12		0.894	—
	DiffCV	15.89 ± 13.62		22.51 ± 11.18		0.200	—
VAmp	M	0.764 ± 0.22	0.796 ± 0.19	0.87 ± 0.14	0.89 ± 0.20	0.049	0.26
	CV	0.16 ± 0.07	0.15 ± 0.06	0.14 ± 0.03	0.13 ± 0.06	0.218	—
	DiffM	6.56 ± 5.36		3.30 ± 2.17		0.077	—
	DiffCV	9.15 ± 6.997		8.05 ± 9.14		0.437	—
Frequency [Hz]	M	13.95 ± 1.36	14.52 ± 1.32	14.19 ± 1.59	14.26 ± 1.10	0.835	—
	CV	0.05 ± 0.03	0.04 ± 0.03	0.04 ± 0.02	0.05 ± 0.03	0.798	—
	DiffM	1.86 ± 2.74		1.54 ± 1.66		0.894	—
	DiffCV	13.65 ± 9.32		9.61 ± 6.68		0.229	—
**MMGpect (n**_**PD**_** = 33; n**_**Con **_**= 42)**		**Less affected**	**more affected**	**le**	**ri**	**Sign. p**	**Effect r**
Slope [°]	M	0.12 ± 0.21	0.26 ± 0.31	0.26 ± 0.34	0.23 ± 0.25	0.594	—
	CV	35.95 ± 130.1	1.52 ± 0.9	11.57 ± 42.0	2.60 ± 2.7	0.432	—
	DiffM	0.42 ± 0.40		0.34 ± 0.27		0.877	—
	DiffCV	17.94 ± 14.98		17.10 ± 13.46		0.900	—
VAmp	M	0.71 ± 0.19	0.68 ± 0.16	0.87 ± 0.16	0.84 ± 0.16	0.000	0.43
	CV	0.13 ± 0.05	0.15 ± 0.07	0.14 ± 0.06	0.14 ± 0.08	0.711	—
	DiffM	6.03 ± 2.73		5.28 ± 3.07		0.204	—
	DiffCV	12.00 ± 10.04		11.95 ± 7.35		0.627	—
Frequency [Hz]	M	13.81 ± 1.2	13.49 ± 1.1	13.77 ± 1.2	14.03 ± 1.3	0.389	—
	CV	0.04 ± 0.03	0.05 ± 0.02	0.05 ± 0.02	0.06 ± 0.05	0.247	—
	DiffM	1.77 ± 1.07		2.04 ± 1.52		0.931	—
	DiffCV	12.02 ± 10.12		13.29 ± 12.05		0.872	—

#### MMG-signals

**Figure 7:**
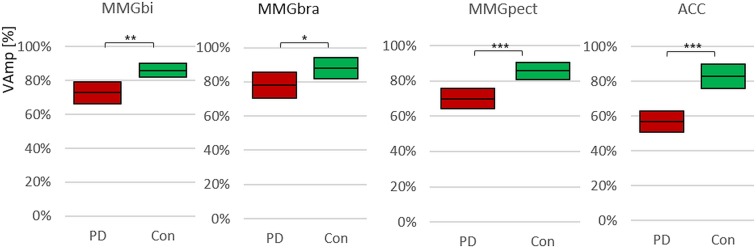
Amplitude variation (VAmp). Displayed are the 95%-confidence intervals of the arithmetic mean of the amplitude variation (VAmp) comparing PD-patients (red) and controls (green) and showing the significantly higher variation of amplitudes in controls in the signals of MMGbi, MMGbra, MMGpect and ACC with *p = 0.049, **p = 0.001 and ***p = 0.000, respectively.

The MMG-signals of biceps brachii and pectoralis major muscles show no significant differences between PD and controls concerning mean frequency and slope (Table 3). The amplitude variation shows significant differences between PD and Con for all MMG signals, highly significant for MMGbi and MMGpect, whereas the MMGbra is just significant (MMGbi: t(76) = 3.52, p = 0.001; r = 0.374; MMGbra: t(55) = −2.017, p = 0.049, r = 0.262; MMGpect: t(71) = 3.965, p = 0.000, r = 0.43), whereby the amplitude variation is higher in controls compared to the PD-group. The 95%-CIs are disjoint for MMGbi, MMGpect and ACC (Fig. 7), the MMGbra shows a decisively lower distinction between PD and Con, however, still is significant. The side asymmetries are not significant in comparing PD and Con (Table 3).

#### ACC-signal

The ACC shows simliar behaviour for the arithmetic mean of slope (n.s.; p = 0.671) and amplitude variation (p = 0.000, r = 0.67; Table 3). Accordingly, the amplitude variation within one trial is significantly higher, whereas the CV of amplitude variation between the trials is significantly lower in controls compared to the PD group (p = 0.002, r = 0.50).

**Figure 8:**
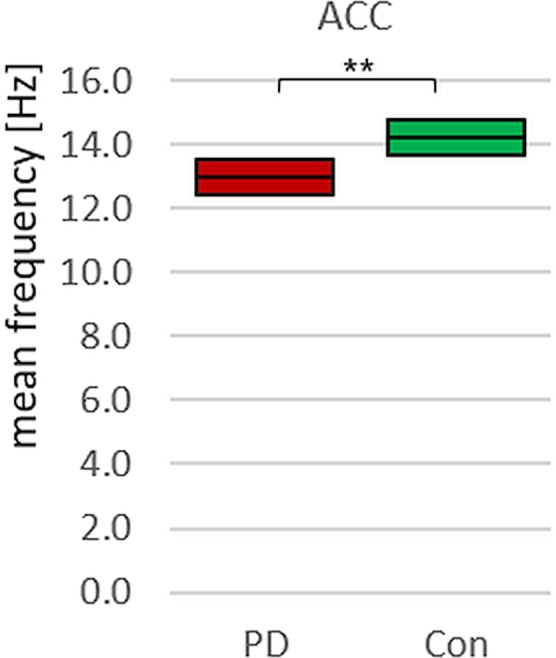
Mean Frequency. Displayed are the 95%-confidence intervals of the mean frequency comparing PD-patients (red) and controls (green), showing the significantly higher mean frequency in controls in the ACC-signal *p = 0.009.

Furthermore, the mean frequency of ACC in PD is significantly lower compared to the controls (p = 0.009, r = 0.43). Although the absolute difference of the mean frequency between PD and Con group amounts only 1.21 Hz, it is significant due to the low coefficient of variation (CV_PD_ = 8% and CV_Con_ = 9%, respectively; Fig. 8). Thereby, the mean frequency of PD is 8.5% lower compared to the mean frequency of Con.

## Discussion

The results show that there might be specific parameters of muscular output that distinguish PD patients without tremor from healthy controls. Before the results are discussed in detail, the methodological limitations are specified.

### Limitations

#### Proportion of gender

Initially, it was planned that both groups (PD and Con) consist of ten female and ten male participants. In the course of the study, it turned out to be more difficult than expected to recruit female Parkinson patients without tremor. Therefore, we decided to accept a difference in gender proportion in the groups.

This could be a potential error source, but since MMG is gender-neutral and the investigated parameters differ not statistically significant with respect to gender, the potential for error is assessed to be minor. The force comparison, which is more relevant with regard to possible influences on the oscillatory behaviour of signals, show no significant difference between groups PD and Con. However, especially in men, the PD group has considerably lower bMVIC (≈ −20 Nm) compared to male controls (p  =  0.125). This could have an influence on signal quality as well as on the amplitude. Since only relative parameters concerning the amplitude were considered, this should not have an influence on the outcome.

#### Setting

When interpreting the results, it has to be taken into account that the participants performed a unilateral task (not considered here) prior to the bilateral task presented here. This could lead to an increased exhaustion and, thus, could influence the signal characteristics. Since both groups performed the same trials prior to the bilateral task, the influence of this should be similar in both groups. But having in mind that for PD patients the preload could relatively be more fatiguing than for healthy controls, the influence on the signals might be higher in PD. This could have potentially enhanced the differences between both groups.

#### Signal quality

One main limitation could be the signal quality. According to Husar^36^ the signal to noise ratio (SNR) of raw signals has to be better than 10 dB for analysing the oscillatory behaviour of data. Therefore, all signals with a lower SNR were excluded. In the beginning, two piezoelectric sensors had to be replaced because of a generally lesser quality than indicated. Furthermore, especially participants with a low bMVIC showed a lower SNR. Since signals with a low SNR were excluded, the quality of the results should not be limited by that fact. However, the sample size is, therefore, reduced in some comparisons.

#### Multiple testing

As usual for explorative studies, which investigate new aspects, several parameters are examined. In the present study, in total 56 comparisons were done. Some authors state that in explorative studies or non-clinical studies, a correction is not mandatory^37–39^. Of course, the results have to be considered with caution due to the explorative character and the small sample size. However, using a correction for multiple testing as the Simes procedure, still ten comparisons stay significant. In the Simes procedure, the *p*-values are firstly sized. Afterwards, the adjusted *p*-values are calculated for each test by ${p}_{i}=\frac{\alpha \ast i}{n}$, for α = 0.05, i = 1,…,n with n = 56. The adjusted *p*-values p_i_ are then compared with the significance p of the performed test. Here, only two formerly significant comparisons turn out to be not significant using the Simes procedure. This is the case for the side asymmetry of the arithmetic mean of the parameter MQ_REL_ (Diff-MQ_REL_) for the pectoralis major muscle (p = 0.014). According to the Simes correction, the *p*-value should be smaller than p_adj_ = 0.0125 for indicating a significant difference. Secondly, *p*-value of the parameter VAmp of the MMGbra in comparing PD and Con (p = 0.049) should be smaller than p_adj_ = 0.011.

In summary, we suppose that the significant differences between the groups are sufficiently large even by considering multiple testing.

### Content-related discussion

The results indicate that PD patients without a resting tremor in medication off phase show differences in some parameters of neuromuscular activity compared to healthy controls during the performed bilateral task. The main results are: 1) Change in power distribution in specific frequency ranges below 12 Hz; 2) a lower amplitude variation in PD; 3) a lower mean frequency in PD for the accelerations generated by the upper extremities, but not for the mechanical muscle oscillations; 4) no difference concerning the slope of amplitude maxima and 5) no statistical difference in side asymmetries except for the parameter MQ_REL_ of MMGpect, which is – after considering multiple testing – not significant anymore. It is assumed that possible side asymmetries are balanced, as the sides influence each other mutually in the bilateral task.

In the following, probable neurophysiological explanatory approaches shall be discussed for the found results. Thereby, the authors are aware of the fact that it is not clear yet, if the results are specific for Parkinson’s disease or if other neurodegenerative diseases would show similar changes. Studies in order to compare different neurodegenerative diseases are mandatory. Further studies must be carried out anyway in order to examine whether the found results are reproducible. The following discussion is based on the assumption that the results will be confirmed by further investigations and are specific for Parkinson’s disease.

Another limitation in the discussion is that almost all investigations concerning neuromuscular oscillations in PD are conducted in patients with a clinical apparent tremor. Only two studies were found that also referred to PD patients without the presence of a tremor.

Milanov investigated the motoneuron activity using the F-wave in patients with different tremor types, including PD with and without tremor^40^. They found no difference concerning the F-wave in patients with and without tremor. In both groups, the motoneuron activity was increased after nerve stimulation compared to controls^40^.

The authors Benninger *et al*. of the second study comparing PD patients with and without tremor investigated morphological changes with a voxel-based morphometry (VBM)^41^. They found a loss of grey matter in the right quadrangular lobe and declive of the cerebellum in PD with tremor compared to without. The cerebellum is involved in several motor control mechanisms. The authors assumed that the loss of grey matter is relevant for the motor symptoms^41^. In the bilateral task of the presented study, the participants have to coordinate the motor action of both upper extremities and the neuromuscular system has to act and react simultaneously. This leads to a more complex motor task compared to a unilateral one. However, the relation of cerebellar grey matter of PD patients without tremor compared to healthy controls is not reported in Benninger *et al*.^41^.

Because of the lack of further studies, the following discussion will relate on investigations in patients with tremor and using other methods; in awareness of the limitations on transferability.

#### Power distribution as a probable indicator for neuropathological processes

The authors hypothesized that in PD the frequency of myofascial oscillations would be shifted in lower frequency ranges. This was assumed because of the usually arising resting tremor of 3–7 Hz in PD patients^42^. These frequency changes were expected to be detectable in a premotor state by means of the mechanical muscular micro-oscillations. However, the parameter MQ_REL_, which points out the proportion of the power in the frequency range of 3 to 7 Hz (I1) to the power in the frequency range of 7 to 12 Hz (I2), is higher in healthy controls compared to the PD group (Fig. 5). This result can arise in three cases: (1) the power in I1 is relatively higher in controls or (2) the power in I2 is relatively lower in controls compared to PD patients – or (3) both. Taking all oscillating signals (MMGbi, MMGbra, MMGpect, ACC) into account, the arithmetic mean of power of I1 amounts approximately 38±3% of the total frequency range of 3 to 12 Hz in controls, whereas in PD the power of I1 amounts approximately 20±4% of the total considered frequency range. Removing the MMGbra from this consideration – since the MMG-signal of the brachialis muscle seems to behave slightly different – the proportions even get clearer: Con: 37±2% vs. PD 18±2%. That speaks for a shift of the power proportion, but, however, not in the expected way. During measurements in the presented specific bilateral setting, PD patients show even a relatively decreased power in the lower frequency band compared to healthy controls. The reason of this apparent contradiction to our hypothesized expectations could be seen in two differences in the present investigation: (1) PD patients without tremor were observed and (2) a novel bilateral setting was applied. Since this was never performed in advance, the exact behaviour of muscular oscillations was not foreseeable.

The mechanical oscillations must be an expression of neuronal activity, which controls motor action. However, MMG does not provide a direct insight into supraspinal processes. The concrete details of the activity in the involved neuronal networks still remain widely unclear^43–45^, but a lot of research is done concerning the oscillations of the subthalamic nucleus as part of the basal ganglia in PD patients with tremor. Especially, the beta band oscillations seem to play an important role in the pathophysiology of PD^21,46–48^, which are rather linked to the symptoms bradykinesia and rigidity^22,49^.

Priori *et al*.^44^ reported a change of lower frequency bands (2–7 Hz, alpha (7–13 Hz) and beta (13–30 Hz)) of the local field potential (LFP) of the subthalamic nucleus (STN) in the course of deep brain stimulation (DBS) in off and on medication state in PD patients. Thereby, the low frequency (2–7 Hz) activity increased after levodopa administration, whereas the low beta (13–20 Hz) activity decreased in the on-state^44^. Thus, the relation of low (2–7 Hz) to beta (13–20 Hz) activity changed in a way that the power ratio of low to high frequencies increased after levodopa administration. In interpreting the diagrams of Priori *et al*.^44^, the power ratio in the band of 3–7 Hz to 7–12 Hz would also increase after L-Dopa administration. These results would rather support the presented findings concerning the power ratio of mechanical motor output under the following assumptions: Firstly, the on-state reflects a more physiological behaviour and secondly, the electrophysiological neuronal activity is transferable to mechanical muscular oscillations.

Recent investigations concentrate also on high frequency oscillations (HFO) of LFP in the STN in DBS^22,23,43^. Thereby, the ratio of slow HFO (~250 Hz) to fast HFO (~350 Hz) was analysed in PD patients comparing the medication off and the medication on state. The ratio increased in medication off during tremor and non-tremor epochs^22,23^. Under medication, the tremor epochs show the same power ratio compared to the medication off, but in epochs without tremor, the power ratio decreased^23^. Thus, faster HFO increased, whereas the slower HFO decreased on medication^22,23^. Hirschmann *et al*.^23^ suppose that the “dopamine-induced shift towards higher frequencies offers relative protection against spontaneous tremor emergence.” (p. 1557). It has to be considered that the shift of power in HFO depends on the used electrode (micro or macro) and its localization in the STN (ventral, dorsal, central)^24^. Furthermore, the question of the physiological behaviour of those HFO in healthy subjects remains. Due to the invasive character, investigations of LFP of the STN are not practicable in healthy controls. Additionally, it is not known, how these neuronal oscillations behave in PD patients without tremor and during an isometric motor task. Nevertheless, the changes of HFO seem to relate to impaired motor processing and could be a functional marker of motor state^21,50^.

These investigations indicate that changes in power distribution of subcortical and cortical oscillations in several frequency ranges occur in PD patients. Due to the complex neurological processes, the changes, of course, cannot be transferred one by one to muscular activity. It is only conjecturable how the muscular oscillations would reflect those changes of brain activity. However, since the interaction of STN and cortex is relevant in motor control^51,52^, it is conceivable that the changes in power distribution of brain activity might be somehow visible in the mechanical output of muscles already in a premotor state.

However, the designs show substantial differences not only concerning the measuring object (brain vs. muscle): In the present investigation, patients without tremor were selected deliberately, since it is decisive to investigate the pathophysiology in premotor state to contribute to the insights into initial changes and early diagnosis. Additionally, in contrast to the above mentioned studies, in which the patients were examined mostly in rest, we investigated the muscular oscillations during a bilateral isometric motor task. Despite the differences, our results suggest that changes in power distribution of mechanical muscle oscillations are present without a manifest tremor. It is not excluded that a complex motor action like the presented particular bilateral task, which requires an interaction and therefore adaptation between left and right side, reflects a shifted subcortical or cortical balance of power in specific frequency bands as was shown by the researchers mentioned above.

The question arises, what is behind the observed change of power ratio in neuromuscular oscillations between low and higher frequency ranges, favouring the activity in higher frequencies? Several authors discuss with regard to beta oscillations that it might reflect a need of faster information processing of motor circuits to compensate the pathological changes^22,46,47,50,52,53^.

#### Reduced variability as sign for impeded motor control

The presented results show a significant decrease in amplitude variation within one trial in PD patients compared to controls concerning MMG and ACC signals. This is interpreted as a reduction in variability of motor control.

Since changes in variability often occur in various pathologies^54–61^ this could be a relevant functional parameter to distinguish PD patients in a premotor state from controls. In general, the change of variation – an increase or a decrease – depends on the considered parameter. Increased variability in PD patients is reported for blood pressure^62^, parameters of gait line as stride duration^63,64^, swing and step time^64^. A decrease in variability in PD patients is found, e.g., in the fundamental frequency of speech^14,65,66^. Since the variation of frequency in speech depends on the thyroarytenoid muscle, this could explain the similar results of muscular oscillations in the here performed bilateral task. In any way, the authors suppose that a particular amount of physiological variability is required in neuromuscular control processes to be able to adapt to external stimuli. In disease, this might be reduced and might reflect impaired neuromuscular control.

The basal ganglia are involved in controlling voluntary movements^52,67^ and, furthermore, the cortical beta activity is widely accepted to be involved in static motor control^52^. Several other stimuli, as sensory or auditory, are also influencing the desynchronisation or synchronisation of alpha and beta activity of several brain areas^68^. Since the present setting involves a bilateral static motor task, it would be conceivable to assume that the causation of the changed variability in PD might lie in a change of the alpha- and/or beta-band of brain activity. The subthalamic and cortical beta-bands usually desynchronise during voluntary actions^48,52^. This could cause an increased variability of muscular oscillations. If this desynchronisation of beta-band does not occur – as found for PD patients in the medication off state^52^ – the variability of the motor output might be reduced. This would support the results of the present study.

Performing a voluntary motor task, a functionally intact cooperation of several neural structures is required, at least of the brain stem, basal ganglia, the motor cortex, premotor cortices, the cerebellum as well as of the spinal cord^69,70^. The neuronal oscillatory processes are not fully understood, neither in healthy persons nor in patients with diseases. In the words of Pfurtscheller and Lopez da Silva: “Neuronal networks can display different states of synchrony, with oscillations at different frequencies”^68^. Therefore, several changes of synchrony and different frequency bands could be relevant in explaining the changed variability of the amplitudes in MMGs and ACC.

The impossibility to measure the basal-ganglia-cortical coupling in healthy complicates the analysis of physiological behaviour of neuronal oscillations. Provided that the muscular output would reflect the behaviour of the supraspinal networks of the blackbox brain, probably, the present setting could facilitate the access to acquire information of healthy persons. Further investigations, of course, remain.

#### Bilateral task for enhancing the effects

The differences of the regarded parameters (especially amplitude variation and power distribution) found between PD and controls were enhanced in the bilateral task compared to a unilateral one (publication in work). This novel approach requires a more complex and demanding sensorimotor control compared to unilateral isometric actions. The generated force of both arms has to be adjusted simultaneously by the brain. Moreover, during this action both sides merge into a mutual dynamic equilibrium characterized by stochastically distributed sinusoidal oscillations of the reaction force and minor motions. This behaviour can only be achieved by a well-functioning coordination of both hemispheres. It is conceivable that this should imply a more challenging effort, especially in subjects with neurological impairments. Symptoms of changed motor control would consequently appear more pronounced.

The effect of the Jendrássik manoeuvre could be another example of this principle. It is known to enhance the H-reflex and tendon reflexes^71–73^ due to a facilitating effect caused by the bilaterally performed manoeuvre. We assume, therefore, that the bilateral task could lead to clearer differences between PD patients and controls compared to the unilateral one. Probably, the pathological changes of neuronal control of muscular activity emerge and dominate therein.

## Conclusion and Outlook

A causality for the lower amplitude variation and lower power ratio of the MMG and ACC signals in PD patients without tremor compared to controls cannot be clarified at the moment. The pathophysiological meaning remains unclear. The comparison of the present findings with investigations of oscillatory brain activity shows several deficiencies, starting from the selection of PD patients (with vs. without tremor) through the different settings (measuring in rest vs. bilateral motor task) and ending with two different objects of observation (brain activity vs. muscular activity).

However, the findings indicate a difference between PD patients without tremor and healthy controls concerning the motor output, which has to be examined further in order to clarify the reproducibility and the clinical relevance of the changes of oscillatory mechanical motor output. Especially, investigations of measuring brain and muscle activity simultaneously would be necessary to clarify the genesis and relevance of the muscular output. Thereby, the synchronisation, variation and power distribution are of special interest. If the findings will be supported by further research, the MMG and ACC measuring could contribute to get further insights into pathophysiology of Parkinsonism and could be evolved into an easy to handle, simple to perform and low cost diagnostic tool. Of course, provided that the specificity for PD would be fulfilled. A suitable wireless handheld device to perform the bilateral task is currently under development (funded by the German federal administration).

## Supplementary information

Supplementary information

## Data Availability

The datasets generated and/or analysed during the current study are available from the corresponding author on request.
