# Supplementary material for: Parkinson patients without tremor show changed patterns of mechanical muscle oscillations during a specific bilateral motor task compared to controls
Source: Sci Rep. 2020 Jan 24;10:1168. doi: 10.1038/s41598-020-57766-5 (PMC6981166; doi:10.1038/s41598-020-57766-5)
Supplement: Supplementary file 1 — Supplementary information [file 41598_2020_57766_MOESM1_ESM.pdf]

Parkinson patients without tremor show changed patterns of mechanical muscle oscillations during a specific bilateral motor task compared to controls

– Supplementary Information –

Laura V Schaefer[1*], Frank N Bittmann[1]

[1] Regulative Physiology and Prevention, Department Sports and Health Sciences, University of Potsdam, Potsdam, Germany

Table 1. Test of normal distribution

Results of Shapiro Wilk test to check the parameters for normal distribution in the groups Con (0) and PD (1) for the single values of each signal MMGbi, MMGbra and MMG pect for left and right side as well as for the relative side asymmetry (Diff).

			Single values of left and right side			Relative side asymmetry (Diff)		
		Group	statistic	df	sign. p	statistic	df	sign. p
MMGbi	MQ_{REL}	0	0.967	38	0.320	0.917	18	0.113
		1	0.964	29	0.400	0.887	13	0.088
	CVQ_{REL}	0	0.791	38	**0.000**	0.938	18	0.270
		1	0.670	29	**0.000**	0.937	13	0.416
MMGbra	MQ_{REL}	0	0.962	30	0.355	0.945	12	0.570
		1	0.957	27	0.311	0.950	11	0.645
	CVQ_{REL}	0	0.955	30	0.234	0.946	12	0.581
		1	0.913	27	**0.026**	0.925	13	0.296
MMGpect	MQ_{REL}	0	0.994	38	0.999	0.922	18	0.140
		1	0.925	27	0.053	0.947	13	0.555
	CVQ_{REL}	0	0.929	38	**0.019**	0.954	18	0.484
		1	0.916	27	**0.031**	0.929	13	0.326

				Single values of left and right side			Relative side asymmetry (Diff)		
			Group	statistic	df	sign. p	statistic	df	sign. p
MMGbi	Slope	M	0	0.746	46	**0.000**	0.885	23	**0.013**
			1	0.616	32	**0.000**	0.733	14	**0.001**
		CV	0	0.483	46	**0.000**	0.913	23	**0.048**
			1	0.632	32	**0.000**	0.945	14	0.483
	VAmp	M	0	0.981	46	0.643	0.950	23	0.299
			1	0.979	32	0.777	0.928	15	0.251
		CV	0	0.914	46	**0.002**	0.866	23	**0.005**
			1	0.943	32	0.092	0.980	14	0.977
	Fre-quency	M	0	0.955	46	0.070	0.899	23	**0.024**
			1	0.974	32	0.606	0.929	14	0.294
		CV	0	0.826	46	**0.000**	0.946	23	0.245
			1	0.944	32	0.095	0.895	14	0.097
MMGbra	Slope	M	0	0.578	30	**0.000**	0.591	12	**0.000**
			1	0.704	27	**0.000**	0.712	13	**0.001**
		CV	0	0.757	30	**0.000**	0.945	12	0.570
			1	0.342	27	**0.000**	0.882	13	0.077
	VAmp	M	0	0.980	30	0.828	0.928	12	0.361
			1	0.980	27	0.858	0.845	13	**0.025**
		CV	0	0.979	30	0.808	0.808	12	**0.012**
			1	0.944	27	0.150	0.872	13	0.055
	Fre-quency	M	0	0.929	30	**0.047**	0.708	12	**0.001**
			1	0.952	27	0.243	0.656	13	**0.000**
		CV	0	0.890	30	**0.005**	0.913	12	0.232
			1	0.861	27	**0.002**	0.890	13	0.098
MMGpect	Slope	M	0	0.870	43	**0.000**	0.883	20	**0.020**
			1	0.890	30	**0.005**	0.746	14	**0.001**
		CV	0	0.196	42	**0.000**	0.885	19	**0.026**
			1	0.199	30	**0.000**	0.873	14	**0.046**
	VAmp	M	0	0.949	43	0.056	0.843	20	**0.004**
			1	0.978	30	0.760	0.820	14	**0.009**
		CV	0	0.971	43	0.335	0.965	19	0.668
			1	0.925	30	**0.037**	0.847	14	**0.020**
	Fre-quency	M	0	0.984	43	0.803	0.734	20	**0.000**
			1	0.955	33	0.183	0.835	14	**0.014**
		CV	0	0.762	42	**0.000**	0.839	19	**0.004**
			1	0.953	29	0.216	0.885	14	0.069
